# Single-cell spatial explorer: easy exploration of spatial and multimodal transcriptomics

**DOI:** 10.1186/s12859-023-05150-1

**Published:** 2023-01-27

**Authors:** Frédéric Pont, Juan Pablo Cerapio, Pauline Gravelle, Laetitia Ligat, Carine Valle, Emeline Sarot, Marion Perrier, Frédéric Lopez, Camille Laurent, Jean Jacques Fournié, Marie Tosolini

**Affiliations:** 1 CRCT, Université de Toulouse, Inserm, CNRS, Université Toulouse III-Paul Sabatier, Centre de Recherches en Cancérologie de Toulouse, Toulouse, France; 2 IUCT-Oncopole, Toulouse, France; 3 Laboratoire d’Excellence Toulouse Cancer, TOUCAN, Toulouse, France

**Keywords:** Spatial transcriptomics, Single-cell, Multimodal analysis, Visualization, Open-source, Freeware

## Abstract

**Background:**

The development of single-cell technologies yields large datasets of information as diverse and multimodal as transcriptomes, immunophenotypes, and spatial position from tissue sections in the so-called ’spatial transcriptomics’. Currently however, user-friendly, powerful, and free algorithmic tools for straightforward analysis of spatial transcriptomic datasets are scarce.

**Results:**

Here, we introduce Single-Cell Spatial Explorer, an open-source software for multimodal exploration of spatial transcriptomics, examplified with 9 human and murine tissues datasets from 4 different technologies.

**Conclusions:**

Single-Cell Spatial Explorer is a very powerful, versatile, and interoperable tool for spatial transcriptomics analysis.

## Introduction

The tissue characterization by single cell RNA sequencing (scRNAseq) technology, spatial transcriptomics (ST), unveils information beyond gene expression profiles and revolutionizes biology and medicine. Today, many packages using R or Python can map cells, score gene signatures, infer cell interactions, and detect spatial patterns [18]. However, computationally frugal visualization of multimodal ST images raises challenges not addressed so far. Most ST platforms [4, 10, 13, 14, 17] yield images of million cells and gigabytes of data which multimodal visualization require overlays with thousands cell scores for thousands gene signatures. So despite the current tools for pre-processing ST data and computing scores, producing such multimodal images remains challenging for (1) the joint availability of spatial coordinates and signature scores, (2) the immediate and tunable overlay of image and signature, (3) the user-friendly environment requested by biomedical users. We previously addressed comparable needs for non-ST scRNAseq datasets with Single-Cell Signature Explorer which, using a mere laptop, projects single cell’s signature scores across UMAP or t-SNE and proposes a slider visualisation of entire databases [8]. Other algorithms, whether command line-based or not, also allow analysis of scRNAseq and CITE-seq datasets [1, 3, 15, 19]. However, we also developed the Single-Cell Virtual Cytometer, which allows such analyses using laptops without command lines. Its interoperability with Single-Cell Signature Explorer enables both phenotyping and cell sorting from numerical data [2, 9]. Since it was not possible to explore ST datasets likewise, here we introduce Single-Cell Spatial Explorer (scSpatial Explorer), a user-friendly and open-source software.

## Materials and methods

### Tissue samples

One human spleen sample was selected and processed following standard ethical procedures (Helsinki Declaration of 1975), after obtaining written informed consent from each donor and approval for this study by the local ethical committee (Comité de Protection des Personnes Sud-Ouest et Outremer II) from the Centre de Resources Biologiques (CRB) collection at IUCT Oncopole CHU Toulouse (DC-2009-989) with a material transfer agreement (AC-2008-820 Toulouse).

### Staining and imaging and spatial transcriptomics

Snap-frozen human spleen sample was embedded in Tissue-Tek O.C.T. (Sakura). $10 \mu m$-thick slices were performed and placed on the Visium spatial slide ($10\times$ Genomics, Visium Spatial Protocols—Tissue Preparation Guide—Rev A), fixed with methanol, and stained with hematoxylin and aqueous eosin (HE). This HE-stained tissue was imaged with a ZEISS inverted microscope under $10\times$/0.3 objective, permeabilized, and reverse transcription was performed on the same slide. Additional examples of ST datasets for human cerebellum, spinal cord and murine brain and kidney were downloaded from the $10\times$ Genomics website.

Second strand synthesis, denaturation, DNA amplification, libraries construction were performed following manufacturer’s instructions ($10\times$ Genomics, Visium Spatial Gene Expression Reagent Kits User Guide—Rev C). The libraries were profiled with the HS NGS kit for the Fragment Analyzer (Agilent Technologies) and quantified using the KAPA library quantification kit (Roche Diagnostics). The libraries were pooled and sequenced on the Illumina NextSeq550 instrument using a High Output 150 cycles kit and the cycles parameters : read 1 : 28 cycles, index 1 : 10 cycles, index 2 : 10 cycles, read 2 : 90 cycles.

### Data preprocessing

**Fig. 1:**
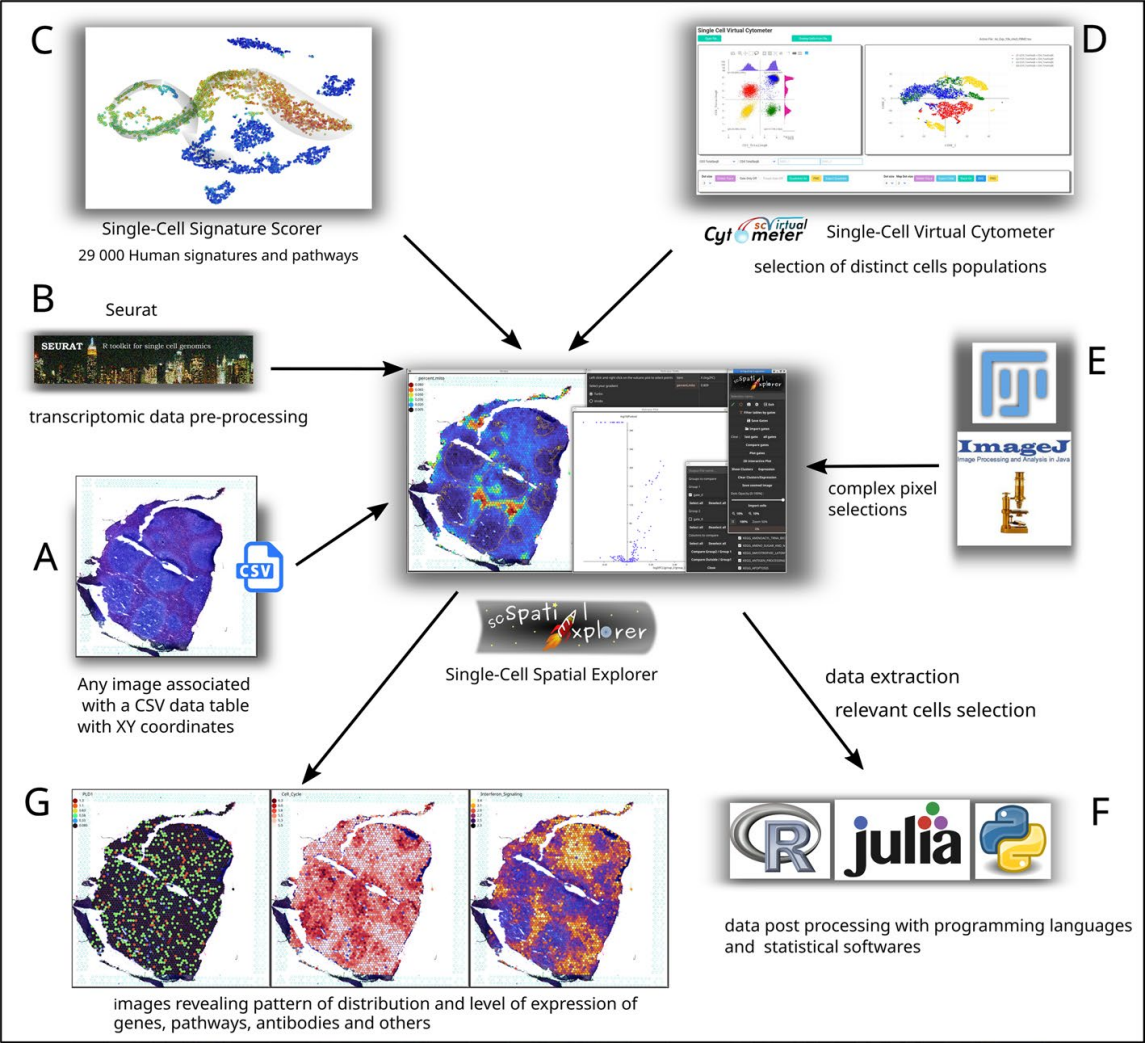
scSpatial Explorer interoperability with any PNG image associated to a table containing spots coordinates and other numeric data (**A**). ST pre-processed data, *e.g.* by Seurat (**B**), can be imported together with signature scores [8] (**C**), to be overlaid with the image. scSpatial Explorer is compatible with Single-cell Virtual Cytometer [9] for spots selections (**D**), it can import/export gates with ImageJ/Fiji format (**E**), and filter an unlimited number of tables from user-defined gates for further processing (**F**). scSpatial Explorer overlays the image with gene or antibodies expressions, or any numerical data. Color and opacity gradients tools allow user to improve scSpatial Explorer overlays (**G**) of image with any numerical data. Licence for logos : R (CC-BY-SA 4.0), Julia (MIT), Python (stating accurately that software [...] is compatible with the Python programming language [...] is always allowed.), FIJI (GPL), ImageJ (public domain), Single-Cell Virtual Cytometer (GNU AFFERO GPL, logo designed by Marie Tosolini), Single-Cell Spatial Explorer (GPL, logo designed by Marie Tosolini)

Both of the HE-stained tissue image and the corresponding FASTQ data were processed using SpaceRanger1.3.0 ($10\times$Genomics) to link the image to the scRNAseq data (Fig. 1A). Prior to their analysis with scSpatial Explorer, the sized image of the sample and its corresponding transcriptome data had to be prepared as follows. First, using a scale factor and image size parameters (“scalefactors_json.json” file), the tissue image and spatial array grid were superimposed to build a sized tissue image (Fig. 1A). Using Seurat 4.1, transcriptomic data were normalized and exported as table (Fig. 1B).

### Spatial ATAC-seq data preprocessing

The murine sample named “ME11 H3K27me3 50um” (GSM5028434) and the Human tonsil sample (GSM528388) were downloaded and data were processed as described in [5] and in [6], respectively, to obtain a spatial Seurat object. Then, genes scores were exported as a matrix, merged with spatial coordinates using the scExplorer Merger [8] and visualized in scSpatial Explorer.

### Single-cell spatial explorer features

The main features of Single-Cell Spatial Explorer are listed in this section. Many of this are also demonstrated in video tutorials.

1. Single-Cell Spatial Explorer is an open source software distributed with a detailed PDF manual and video tutorials.
2. Single-Cell Spatial Explorer has a graphical interface, it is ready to use, pre-compiled binaries are available for GNU/Linux, MacOS, Windows, no installation required.
3. Cross-platform : GNU/Linux, Mac and Windows (the graphical interface and the software are coded in pure Go)
4. Low memory usage. Single-Cell Spatial Explorer memory usage is shown in Additional file 1: Fig. S1.
5. Compatible with any PNG image associated with any data file (text format with tab separator) containing at least the spots tags and the spots XY coordinates of the image.
6. Compatible with any numeric data : gene expression, pathway scores, antibody staining level etc...
7. An unlimited number of gates can be drawn in the microscopy image or in the 2D interactive plot.
8. Import/export gates in ImageJ/Fiji format (Fig. 3).
9. Extraction of spots and sub-tables delimited by the gates on an unlimited number of tables. Exportation is done in TAB separated files for interoperability.
10. 2D plots of the spots inside the gates with any XY coordinates : t-SNE, UMAP, gene expression, pathway scores, antibody staining level etc... (Fig. 4)
11. Interactive 2D plot to show the selected spots on a t-SNE, UMAP or any other coordinates on the image and to filter the data tables into sub-tables (Fig. 4).
12. Cluster display with 3 color gradients, custom color palette, custom dot opacity and custom dot size. The option “Shuffle color” option allows to change color positions on the map, leading to almost 2 billions of possible images with 12 clusters.
13. Display any kind of cell expression (genes, pathways, antibodies...) with 8 preset gradients, custom gradient, custom legend color, dot opacity and custom dot size. The gradients are simple two colors maps and rainbow colors maps Turbo, Viridis and Inferno to allow user to optimize visualisation.
14. The Min/Max intensity sliders allow user to tune cluster or expression contrast or remove artefacts due to outliers. This important option is unique to scSpatial Explorer.
15. The expression opacity gradient can be tuned with min/max threshold.
16. Slide show to review many cell expression maps without need of repetitive clicks. This feature can be seen in https://www.youtube.com/watch?v=mId538e5JDk at 1’40” and https://www.youtube.com/watch?v=dqudL36Dg1M at 7’55”.
17. Screenshot or native resolution image exportation.
18. Import and display an unlimited number of dots lists by repetitive click on the “import cells” button. The format is directly compatible with Single-Cell Virtual Cytometer [9].
19. Comparison of two groups of gates together across the whole dataset.
20. Comparison of one group of gates against all the remaining spots.
21. Draw an interactive volcano plot after gate comparison (Fig. 3).
22. Plot cell expression of a selected dot in the volcano plot (Fig. 3).
23. Export volcano plot image and the corresponding data table.
24. Image zoom 10–200%

### Minimal computer configuration

Single-Cell Spatial Explorer has been developed on GNU/Linux Manjaro (XFCE desktop) on a computer with an Intel ©Core$^{\textrm{TM}}$ i5-3470 CPU @ 3.20 GHz with 8 GB RAM, a 7200 rpm hard disk, a video card Nvidia GeForce GT630 and a HD display with a resolution $1920 \times 1080$. This is a comfortable hardware configuration for a small data set of about 3000 spots.

Although it is possible to use Single-Cell Spatial Explorer on a laptop, we recommend to use a HD display for a more comfortable experience. We do not recommend to use two screens with different resolutions or display zooming.

### Code availability

scSpatial Explorer was developed in Go using the graphical library Fyne. Precompiled static binaries are available for Linux, Mac and Windows. Files can be accessed at GitHub scSpatial Explorer web page.

## Results and discussion

### Single-cell spatial explorer

Single-Cell Spatial Explorer (scSpatial Explorer) is a software for easy analysis of data associated with image and spatial coordinates. It allows, in a very simple way, to visualise features, select region of interest (ROI) and to perform differential analysis in interactive way, draw dot plots of spots, select spots on the image to visualize this on dot plot, or vice versa select spots in dot plot to visualize this on the image. To facilitate data exploration, scSpatial Explorer is compatible with ROI defined with ImageJ/Fiji, cells selected with Single-Cell Virtual Cytometer [9], and any type of data imported as a tab-separated file (Fig. 1). It has many features allowing spots characterisation, selection, exportation and data filtering. It can display quantitative (gene expression, gene sets scores...) and qualitative (clusters) data on a microscopy image. scSpatial Explorer can also filter many tables for selected cells in one click. Any figure, result, table or ROI created in scSpatial Explorer can be exported.

scSpatial Explorer is a software with a graphical interface with two main windows : the microscopy image is displayed in one window and a toolbox in another one. The graphical interface is shown in the center of Fig. 1 and screenshots are shown in GitHub. The software is easy to install and can be run without any programming skills on any computer, and comes with a detailed documentation and video tutorials. scSpatial Explorer has been developed with interoperability as a priority, especially with Seurat [16], Single-Cell Signature Explorer [8] and Single-Cell Virtual Cytometer [9], leading to a very versatile and powerful solution (Fig. 1).

**Fig. 2:**
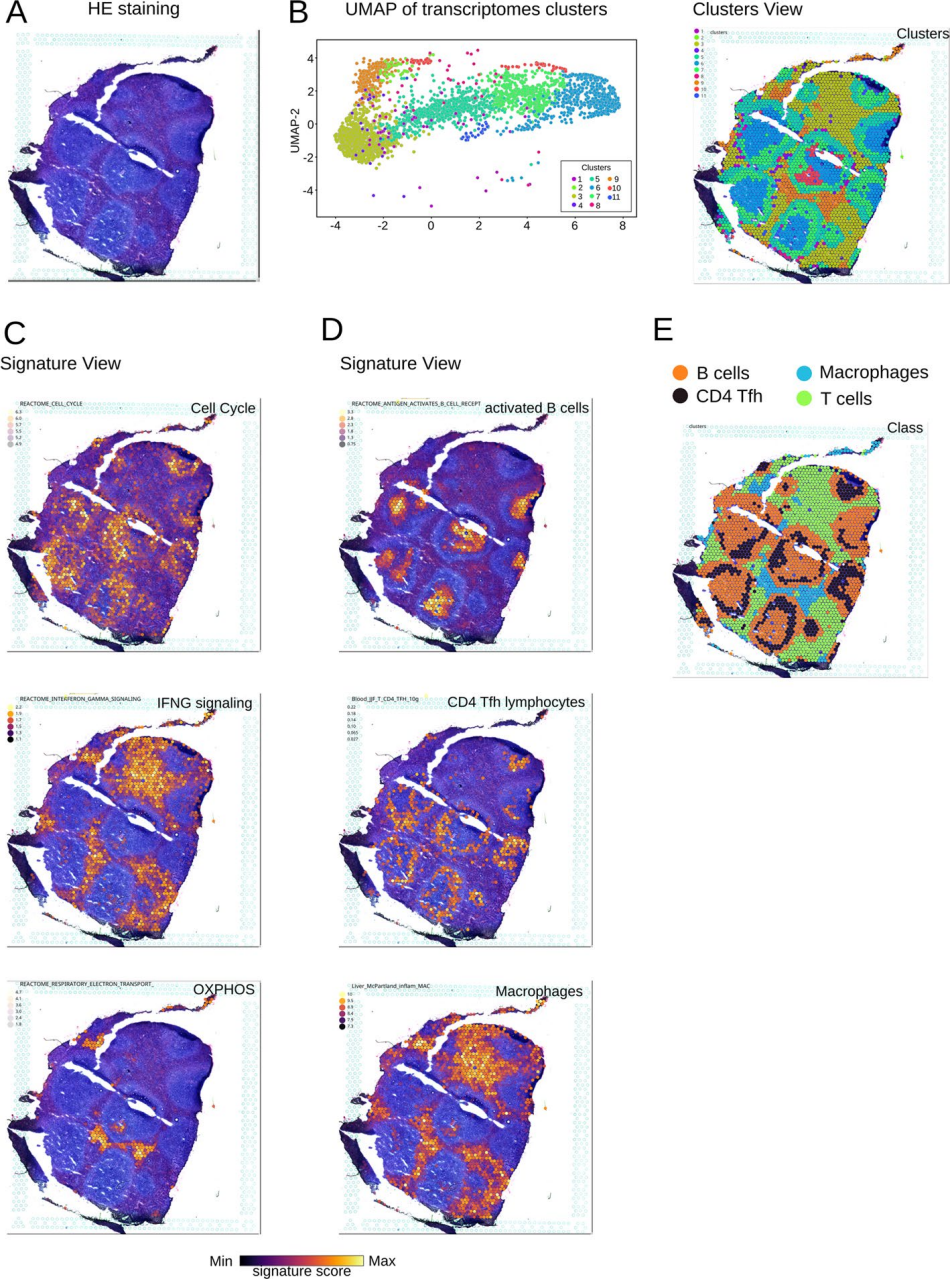
Spleen sample prepared with $10\times$ Genomics Visium and analysed using scSpatial Explorer. Image of hematoxylin eosine staining of a human spleen sample (**A**, **B**): UMAP plot of transcriptomic clusters from the corresponding dataset and spatial overlay of these clusters (same color code) on the tissue image. The Signature View mode (**C**, **D**) overlays the tissue image with the spot’s enrichment scores for selected gene signatures (’Inferno’ gradient heatmap), here showing follicular areas and peri-follicular associated to functions (**C**) and cell types (**D**). The transcriptomic clusters were finally summarized (**E**) into 4 main cell classes: B cells (orange), macrophages (blue), CD4 Tfh (black) and T cells (green)

It overlays on a microscopy image either the quantitative data (e.g. ST tables) shown as color gradient heatmaps or qualitative data (e.g. cluster or categories) delineated using discrete color palettes (Fig. 2). There are 8 different color gradients available. The transparency of color points can be adjusted in two different way: The same transparency for all the spots, or a gradient of transparency based on the feature value (Fig. 2). For transparency gradient, the minimal and maximal values are adjustable.

**Fig. 3:**
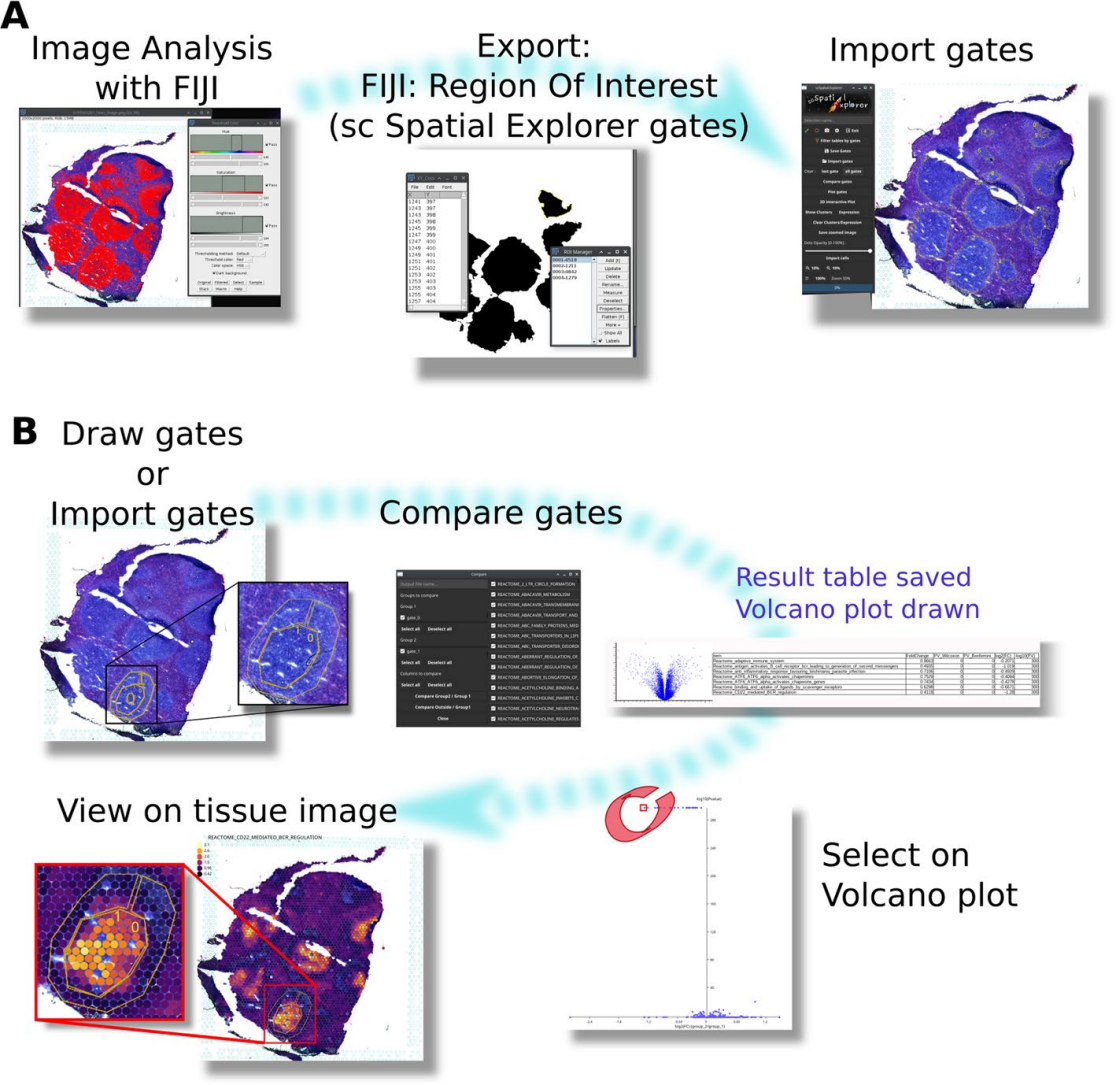
A complex gate pattern was obtained by image analysis. Using ImageJ/Fiji tools, Region Of Interest (ROI, here splenic follicles) were defined, exported and imported in Single-Cell Spatial Explorer (**A**). Gates previously defined with ImageJ/Fiji were compared in Single-Cell Spatial Explorer on the Reactome pathway ($> 2500$ pathways) scores computed in Single-Cell Signature Explorer [8], resulting in a *P* value vs fold change table and volcano plot (**B**). On the volcano plot, a dot was selected and the corresponding pathway (’CD22-mediated BCR regulation’) visualized on the tissue image

**Fig. 4:**
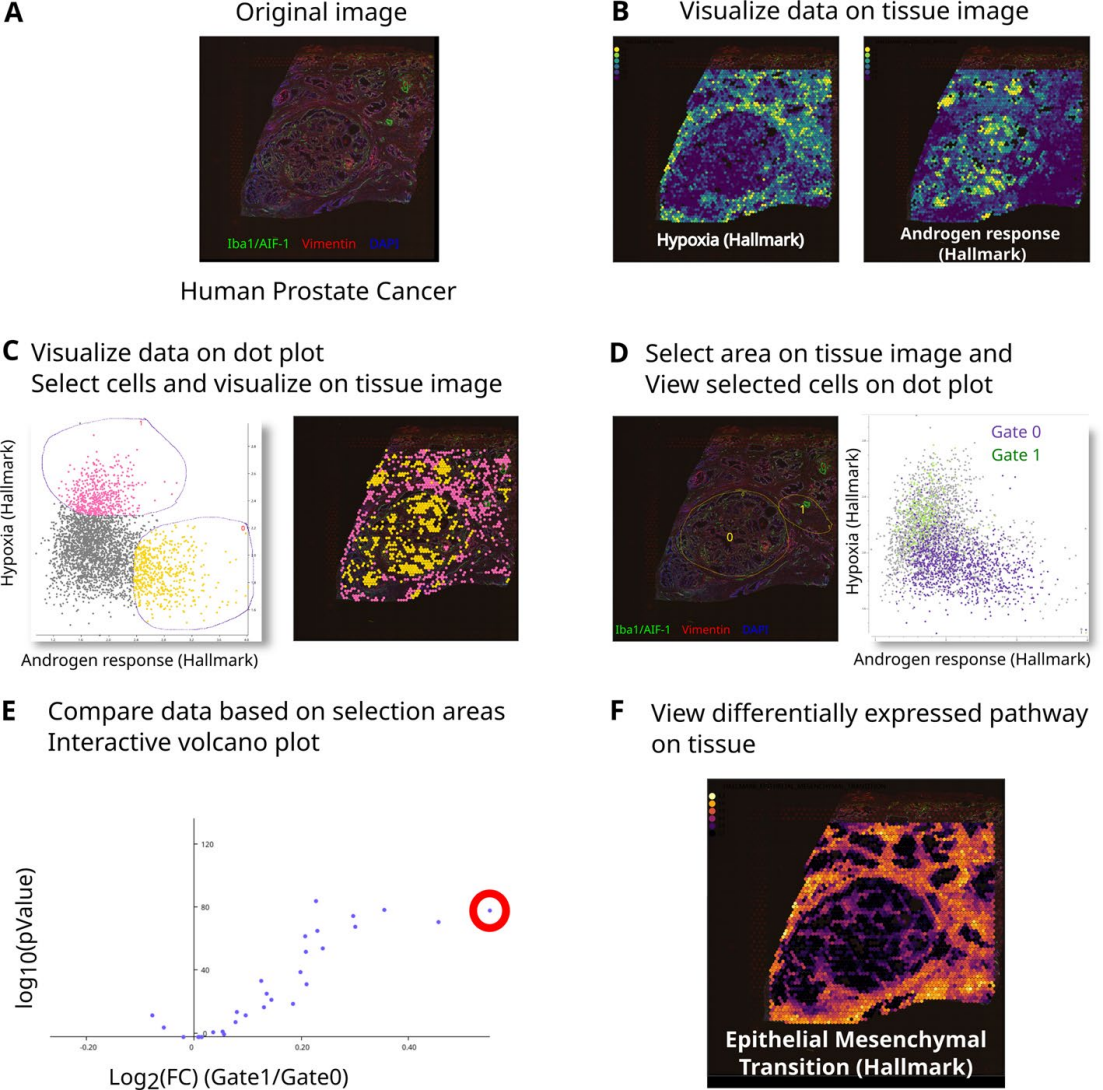
The human prostate cancer dataset was downloaded from $10\times$ Genomics website. 10x Genomics website. The tissue was stained by immunofluorescence using antibodies Iba1/AIF-1 (green),Vimentin (red), DAPI (blue) (**A**). Data were normalized using Seurat [1], exported and Hallmark pathways were scored using Single-cell Explorer Scorer [8]. Using Single-Cell Spatial Explorer, scores from hallmark pathways (hypoxia, left; Androgen Response right) are visualized (**B**). **C** On the “Hypoxia”/“Androgen Response” dot plot, spots were selected (Hypoxia$^+$AndrogenResponse$^-$ in pink and Hypoxia$^-$AndrogenResponse$^+$ in yellow) and visualized on the tissue image. **D** Area from the tissue image were selected and spots from this area were visualized on the “Hypoxia”/“Androgen Response ” dot plot (top) or compared using all the hallmark pathways resulting in a table and a volcano plot (**E**). Interesting dot can be selected (red circle in **E**) and the corresponding data visualized on the tissue image (**F**)

It allows to gate any cell population delineated either by a ROI from the image or by a scatterplot (Figs. 3, 4). As mentioned above, a ROI can also be imported from ImageJ/Fiji after an advanced analysis. An unlimited number of data tables containing XY image coordinates can be filtered by gates, which can be further compared or plotted with any XY coordinates. For differential analysis, each gate can be added (or not) in group 1 or in group 2. scSpatial Explorer allows to compare group 1 to group 2 or group 1 to all the none-group 1 spots. Gate comparison displays an interactive volcano plot (Fold-Change *v.s.* *P* value) (Mann-Whitney U test corrected by Bonferroni) from which dots can be selected to get the corresponding expression data and visualize them on the image (see Fig. 3).

scSpatial Explorer is compatible with any PNG image and any data table (TAB separated files) containing XY image coordinates. Thus, the user can apply scSpatial Explorer at any level of his own analytic pipeline. For example, after raw data pre-processing, scSpatial Explorer can import gene or antibody staining level, as well as pre-computed signature scores (e.g. from MSIgDB [7, 8], see Fig. 2). Cell populations gated with Single-Cell Virtual Cytometer [9] can also be imported for a flow cytometry-like analysis and unlimited sub-gatings. scSpatial Explorer can leverage ImageJ/Fiji image processing capabilities for image analysis, by macro-programming and gates importation [11, 12] (see Fig. 3A). Sub-tables, cell names and gates coordinates can be exported in CSV format for further processing.

Comparison with existing ST visualization softwares (Supplementary discussion) positioned scSpatial Explorer as a unique user-friendly tool.

### Example of ST analysis

Below we exemplified ST analysis of a human spleen tissue produced by Visium technology ($10\times$ Genomics). The transcriptome data were visualized by Uniform Manifold Approximation and Projection (UMAP) (Fig. 2A), and the enrichments of all signatures fromMSIgDB were scored by Single-Cell Signature Scorer [8] with the pipeline illustrated in Additional file 1: Fig. S2. Then, distinct exploration modes available on scSpatial Explorer were used.

The ‘Cluster view’ mode overlays the sized tissue image and transcriptomic clusters delineated by a color palette (Fig. 2). Here, the overlay of the spleen image with 11 transcriptomic clusters revealed a follicular pattern yet visible on the HE-stained tissue (Fig. 2A), and the strictly follicular localisation of Clusters 2, 5–8, 10, 11 (Fig. 2B). The ‘Expression view’ mode overlays the tissue image with genes expressions, antibodies staining level or enrichment scores. The user selects any signature from a large collection of curated or user-defined signatures. For example, the follicular areas appeared strongly mitotic while their periphery expressed high level of IFN-signalling and mitochondrial respiration (Fig. 2C). Lineage-specific genesets identified regions with a high proportion of antigen-activated B lymphocytes maturing in the follicles, peripheral rings of a high proportion of follicular helper CD4 T cells, and a macrophage-rich stroma (Fig. 2D). This spatial organization reflected the antibody-producing function of splenic germinal centers. Negative control signatures (randomly selected genes [8]) yield homogeneous images without pattern (data not shown) whereas curated signatures produced highly informative images. In its ’slider’ expression mode, scSpatial Explorer allows rapid screens of collections of signatures (see section “Single-Cell Spatial Explorer features”, item 16). Here, it pinpointed among others: germinal centers, follicular plasmablasts, T lymphocytes, natural killers, dendritic cells, myeloid cells, Hallmarks such as ’G2M checkpoint ’, ’Unfolded Protein Response’, and target of the CEBP2, HSD17B8, SKIL, and ZNF407 transcription factors. Finally, this information allowed to summarize the 11 clusters into 4 main cell classes: intra-follicular B cells and CD4 Tfh, stromal T cells and macrophages (Fig. 2E).

scSpatial Explorer harnesses the image processing developments. To characterize the molecular hallmarks of spatially-defined spots, it can import gates with the same CSV polygon format as ImageJ/Fiji ROI (see Supplementary information). Any cell-delineating pattern defined by image analysis can be imported likewise for analysis by scSpatial Explorer. Gates can also be directly drawn on the image using the lasso or polygon tools. Here, the transcriptomes from an intra-follicular gate and a peri-follicular gate were compared (Fig. 3). This comparison returns a Fold Change and *P* value table, and an interactive volcano plot for the table columns selected by the user. Then, selection of any dot displays expression of the corresponding signature as a heatmap overlay with the image. Here, the human splenic germinal centers up-regulated several signatures such as ’CD22-mediated BCR regulation’ and ’Mitosis’ reflecting BCR-mediated selection of antigen-specific B lymphocytes.

Beyond identifying the transcriptome hallmarks of spatially-delineated spots, scSpatial Explorer indeed allows to localize any image spots selected by transcriptome criteria. For example, from a human prostate cancer ST dataset, spots gated in a scatterplot of “Hypoxia VS Androgen Response” signatures are instantly mapped within the IF image (Fig. 4). Such gates can also be delineated from scatterplots of single gene expressions, immunophenotypes, or any other numerical values from multimodal datasets.

Thanks to the diversity of gene signature on database like MSIgDB [7], different cells type could be identified such as human Glutamatergic or GABAergic neurons (Additional file 1: Fig. S3), glial cells, oligodendrocytes or astrocytes cells (Additional file 1: Fig. S4), murine endothelial cells or nephron progenitor cells (Additional file 1: Fig. S5), as well as behavioural hallmarks (Additional file 1: Fig. S6).

As scSpatial Explorer is versatile, it can process images from HE (Additional file 1: Figs. S3, S5, S6) or IF experiments (Fig. 4, Additional file 1: Fig. S4). Data could come from diverse type of experiment such as transcriptomic but also ATAC-seq (Additional file 1: Fig. S7). In this case, data were processed as described in [5, 6], genes scores were exported as a matrix, merged with spatial coordinate using the scExplorer Merger [8] and analysed in scSpatial Explorer. The results are similar to those shown in [5, 6]. Transcriptomic experiments could arise from transcriptomic dataset yielded from different technologies and platforms as illustrated with $10\times$ Visium (Figs. 2, 3, 4, Additional file 1: Figs. S3–S6) or CoxMx (Additional file 1: Fig. S8).

## Conclusion

scSpatial Explorer is a very powerful, versatile, and interoperable tool for ST analysis. It rapidly overlays the tissue image with quantitative metrics, such as multigene signature scores. It harnesses a memory-efficient approach (Additional file 1: Fig. S1) to the visualization needs of a large audience of biomedical scientists without advanced analytic and programming skills, packages, libraries, and wrappers. We forecast that when reaching the true single cell resolution level, the next generation of ST will benefit even more of scSpatial Explorer and its interoperability, notably with emerging frameworks of spatial pattern meta-analyses.

## Availability and requirements

Project name: Single-cell Spatial Explorer Project home page: https://github.com/FredPont/spatial Operating system(s): Linux, Windows, MacOS Programming language: Go https://go.dev/ Other requirements: to build from source, the prerequisites of the Fyne graphical toolkit (https://developer.fyne.io/started/) are required License: GNU GPL Any restrictions to use by non-academics: restrictions of the licence GNU GPL

## Supplementary Information

**Additional file 1: Figure S1.** Single-Cell Spatial Explorer memory usage. **Figure S2.** Pipeline used to display biological functions in Single-Cell Spatial Explorer. **Figure S3.** Example using a human cerebellum dataset ($10\times$ technology). **Figure S4.** Example using a human spinal cord dataset ($10\times$ technology). **Figure S5.** Example using a mouse kidney dataset ($10\times$ technology). **Figure S6.** Example using a mouse brain dataset ($10\times$ technology). **Figure S7.** Analysis of spatial ATAC-seq experiment. **Figure S8.** Spatial analysis of lung tissue by CoxMx technology. **Table S1.** Spatial transcriptomics softwares comparison Supplementary discussion and references: Memory footprint, Compatibility with ImageJ/Fiji and Comparison with existing softwares.

## Data Availability

The software Single-cell Spatial Explorer is available on github The human prostate cancer dataset was downloaded from 10x Genomics website (https://www.10xgenomics.com/resources/datasets/human-prostate-cancer-adjacent-normal-section-with-if-staining-ffpe-1-standard-1-3-0). The human cerebellus dataset was downloaded from 10x Genomics website (https://www.10xgenomics.com/resources/datasets/human-cerebellum-whole-transcriptome-analysis-1-standard-1-2-0). The human spinal cord dataset was downloaded from 10x Genomics website (https://www.10xgenomics.com/resources/datasets/human-spinal-cord-whole-transcriptome-analysis-stains-dapi-anti-snap-25-anti-gfap-anti-myelin-cn-pase-1-standard-1-2-0). The mouse kidney dataset was downloaded from 10x Genomics website (https://www.10xgenomics.com/resources/datasets/adult-mouse-kidney-ffpe-1-standard-1-3-0). The mouse brain dataset was downloaded from 10x Genomics website (https://www.10xgenomics.com/resources/datasets/adult-mouse-brain-ffpe-1-standard-1-3-0). The CoxMx technology downloaded from Nanostring website (https://nanostring.com/products/cosmx-spatial-molecular-imager/ffpe-dataset/) The spatial ATAC sample “ME11 H3K27me3 50um” (GSM5028434) and Human tonsil sample (GSM528388) were downloaded from geodatasetwebsite (https://www.ncbi.nlm.nih.gov/gds) The datasets of a human spleen tissue analysed during the current study are available from the corresponding author on reasonable request.

## Abbreviations

BCR: B cell receptor
CITE-seq: Cellular Indexing of Transcriptomes and Epitopes by sequencing
FC: Fold change
GO-BP: Gene ontology-biological process
HE: Hematoxylin and eosin
IF: Immunofluorescence
MSIgDB: Molecular signature data base
ROI: Region of interest
scRNAseq: Single cell RNA sequencing
scSpatial Explorer: Single-cell spatial explorer
ST: Spatial transcriptomics
t-SNE: t-Distributed stochastic neighbour embedding
UMAP: Uniform manifold approximation and projection
