# Supplementary material for: Single-cell spatial explorer: easy exploration of spatial and multimodal transcriptomics
Source: BMC Bioinformatics. 2023 Jan 27;24:30. doi: 10.1186/s12859-023-05150-1 (PMC9881287; doi:10.1186/s12859-023-05150-1)
Supplement: Supplementary file 1 — Additional file 1: Figure S1. Single-Cell Spatial Explorer memory usage. Figure S2. Pipeline used to display biological functions in Single-Cell Spatial Explorer. Figure S3. Example using a human cerebellum dataset ($10\times$10× technology). Figure S4. Example using a human spinal cord dataset ($10\times$10× technology). Figure S5. Example using a mouse kidney dataset ($10\times$10× technology). Figure S6. Example using a mouse brain dataset ($10\times$10× technology). Figure S7. Analysis of spatial ATAC-seq experiment. Figure S8. Spatial analysis of lung tissue by CoxMx technology. Table S1. Spatial transcriptomics softwares comparison Supplementary discussion and references: Memory footprint, Compatibility with ImageJ/Fiji and Comparison with existing softwares. [file 12859_2023_5150_MOESM1_ESM.pdf]

Supplementary Information

Single-Cell Spatial Explorer: Easy exploration of spatial and multimodal transcriptomics

Authors:

Frédéric Pont, Juan Pablo Cerapio, Pauline Gravelle, Laetitia Ligat, Carine Valle, Emeline Sarot, Marion Perrier, Frédéric Lopez, Camille Laurent, Jean Jacques Fournié, and Marie Tosolini.

Supplementary figures and table

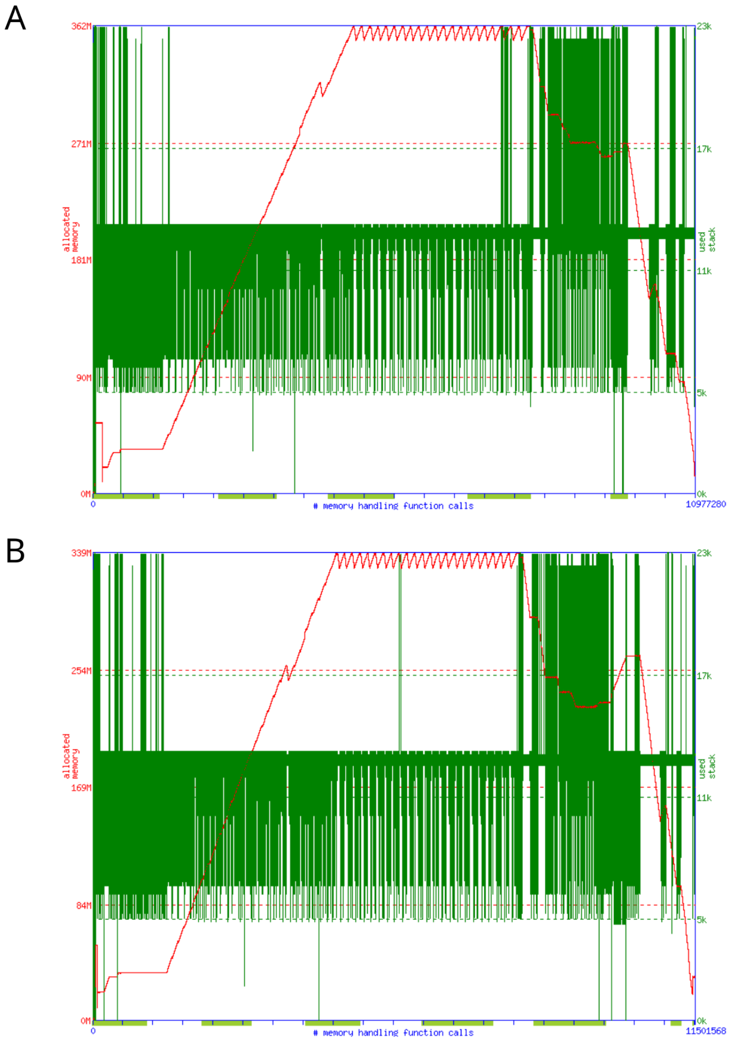

Supplementary Figure 1. Single-Cell Spatial Explorer memory usage. Output of memusage memory profiling software on a entry level computer. Operating system : GNU Linux Manjaro Xfce, Intel(R) Core(TM) i5-3470 CPU @ 3.20GHz with 8 GB RAM. The session monitored contains a cluster plot, followed by 50 expression plots, a volcano plot and 3 min pause before closing the software. A) Single-Cell Spatial Explorer session with a 2798×202 dataframe, 2000x2000 . 362 MB max allocated memory. B) Single-Cell Spatial Explorer session with a 2798×9885 dataframe, 2000x2000 pixels image. 339 MB max allocated memory. Red : allocated memory. Green : stack usage.

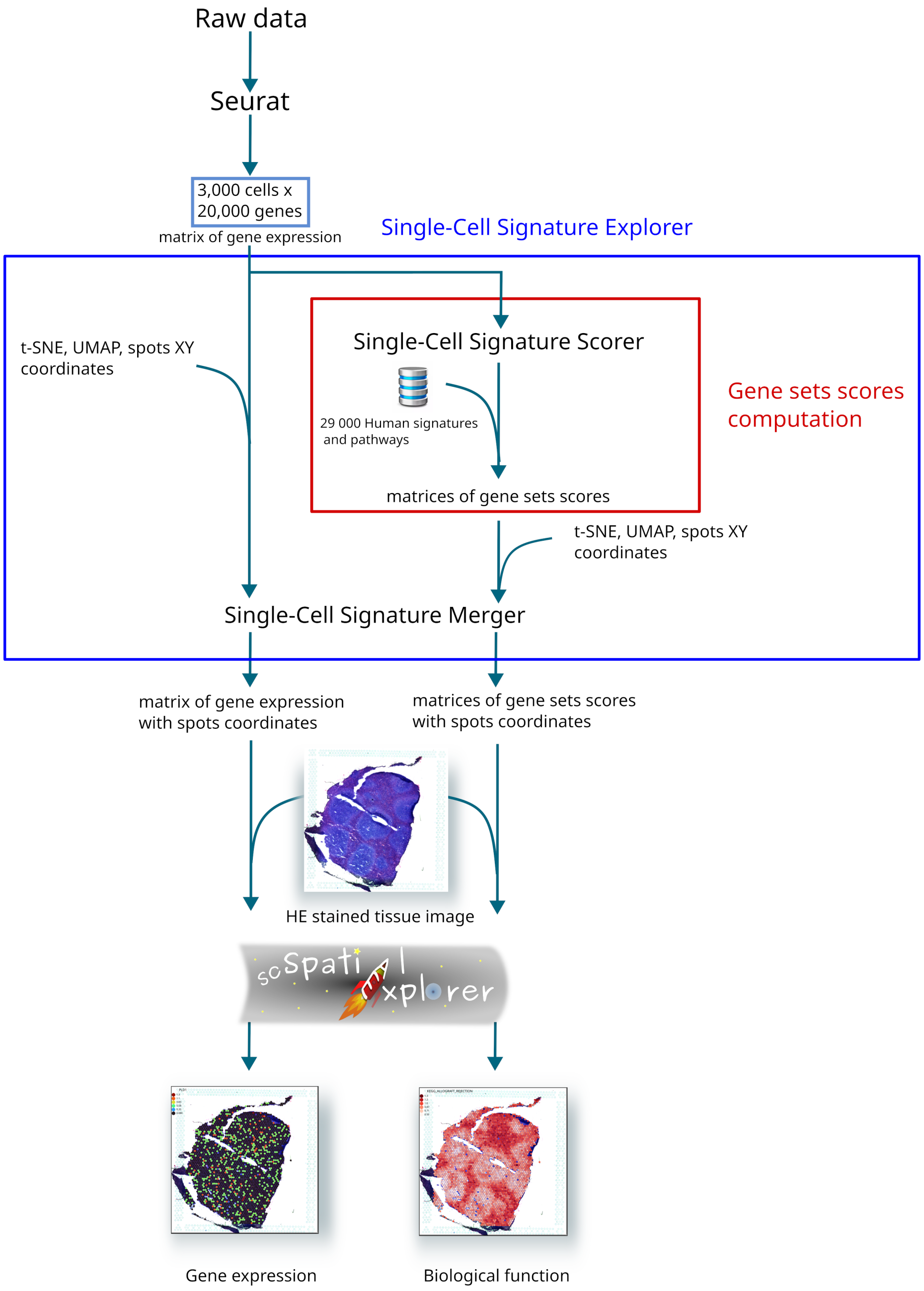

Supplementary Figure 2. Pipeline used to display biological functions in Single-Cell Spatial Explorer. Spatial raw data were normalised in Seurat. UMAP and t-SNE spots coordinates were computed in Seurat and spots XY coordinates in the microscopy image were obtained in SpaceRanger1.3.0 (10xGenomics) . The normalized gene expression matrix was imported in Single-Cell Signature Scorer[1] to obtain matrices of gene set scores. Gene sets extracted from MSIgDB[2] were downloaded here. Spots XY coordinates on the microscopy image as well as t-SNE and UMAP dots coordinates were merged with the matrices of gene sets scores and gene expression using Single-Cell Signature Merger[1]. These matrices were then directly imported in Single-Cell Spatial Explorer to visualize gene expression or biological functions as color maps. UMAP and t-SNE dots coordinates were used to characterize area selected on the microscopy image or to characterize spots on a 2D plot as illustrated in figure 4.

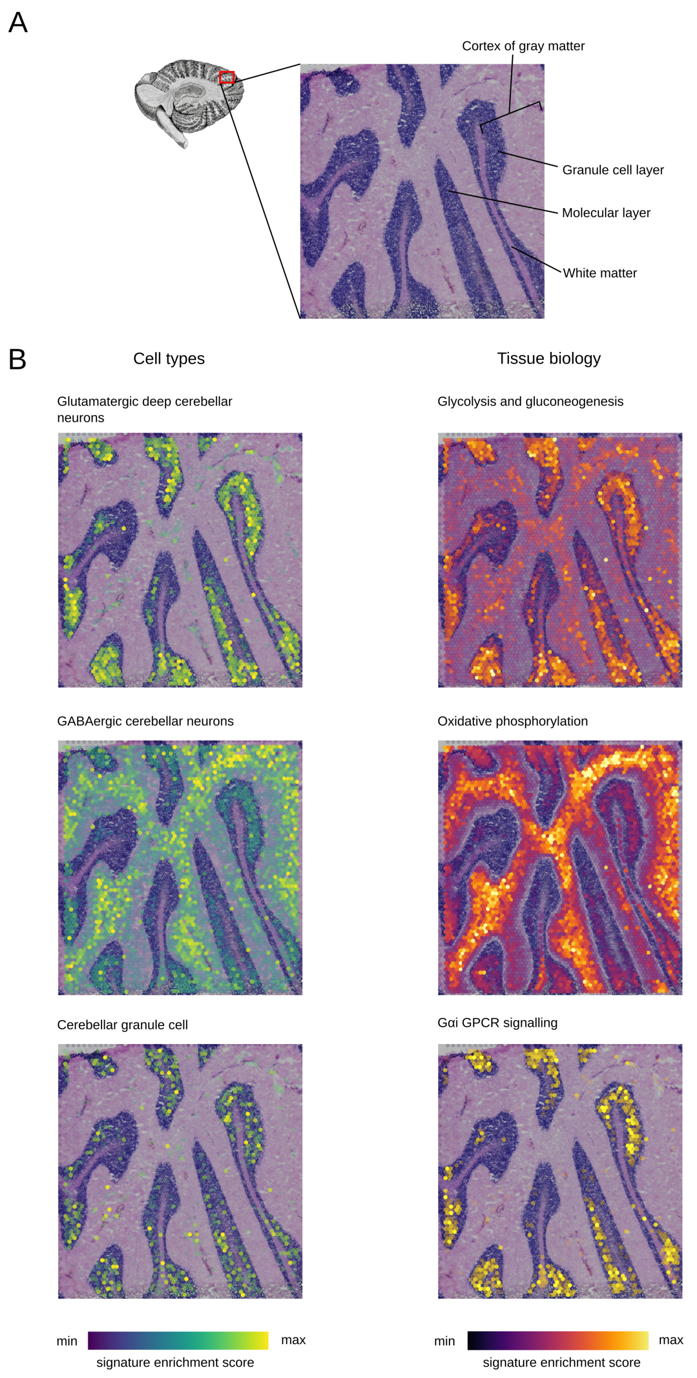

Supplementary Figure 3. The human cerebellus dataset was downloaded from 10x Genomics website. The tissue was stained with hematoxylin and aqueous eosin (HE) (A). Data were normalized using Seurat[3], and pathways from MSIgDB[2] were scored using Single-cell Explorer Scorer[1]. Using Single-Cell Spatial Explorer, signatures of cell types (Viridis gradient heatmap) and tissue biology (Inferno gradient heatmap) are visualized through a min-max scale and opacity threshold (B).

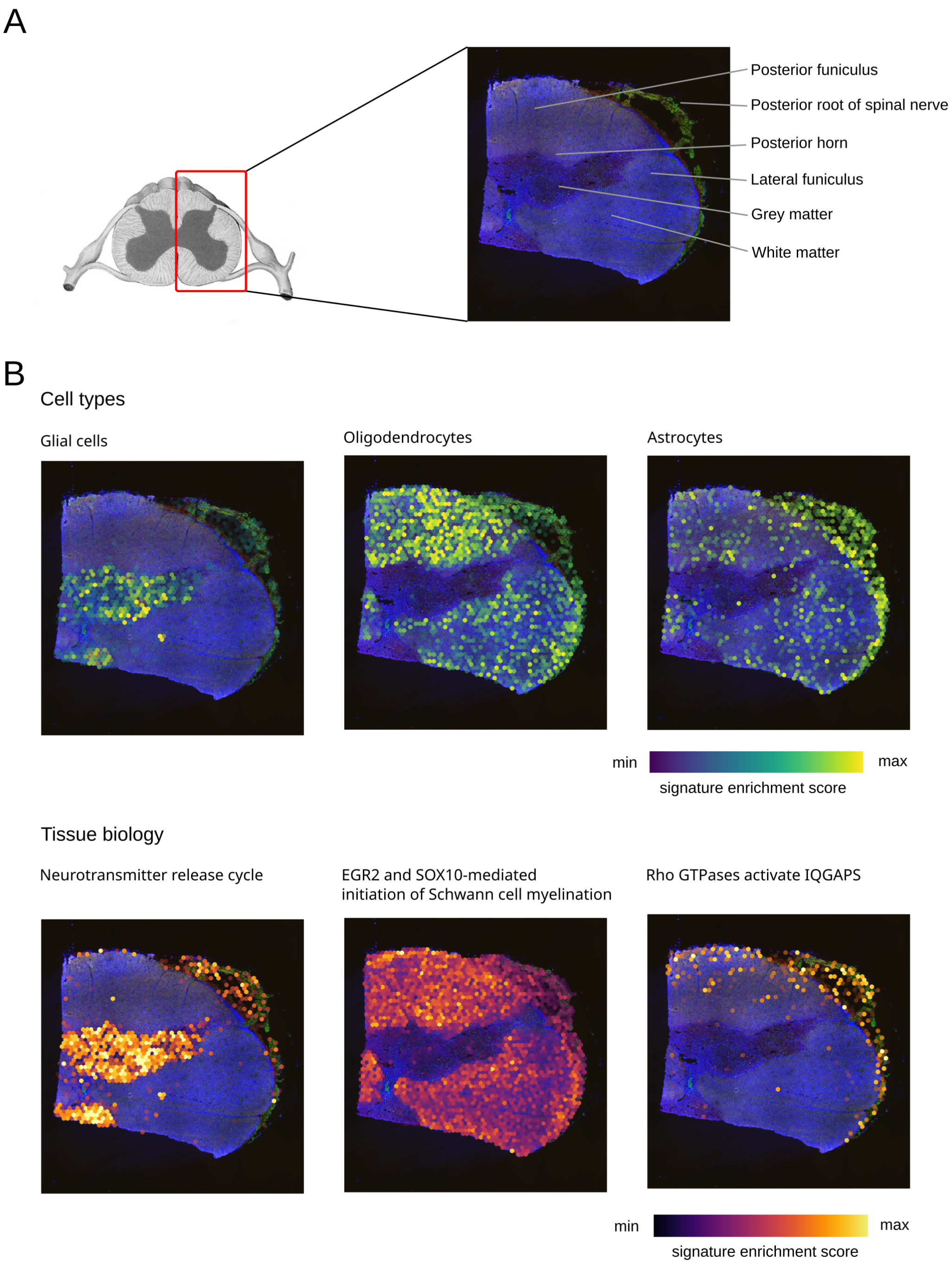

Supplementary Figure 4. The human spinal cord dataset was downloaded from 10x Genomics website. The tissue was stained by immunofluorescence using antibodies Anti-SNAP25 (green), Anti-GFAP (pink), Anti-Myelin CNPase (red), DAPI (blue) (A). Data were normalized using Seurat[3], and pathways from MSIgDB[2] were scored using Single-cell Explorer Scorer[1]. Using Single-Cell Spatial Explorer, signatures of cell types (Viridis gradient heatmap) and tissue biology (Inferno gradient heatmap) are visualized through a min-max scale and opacity threshold (B).

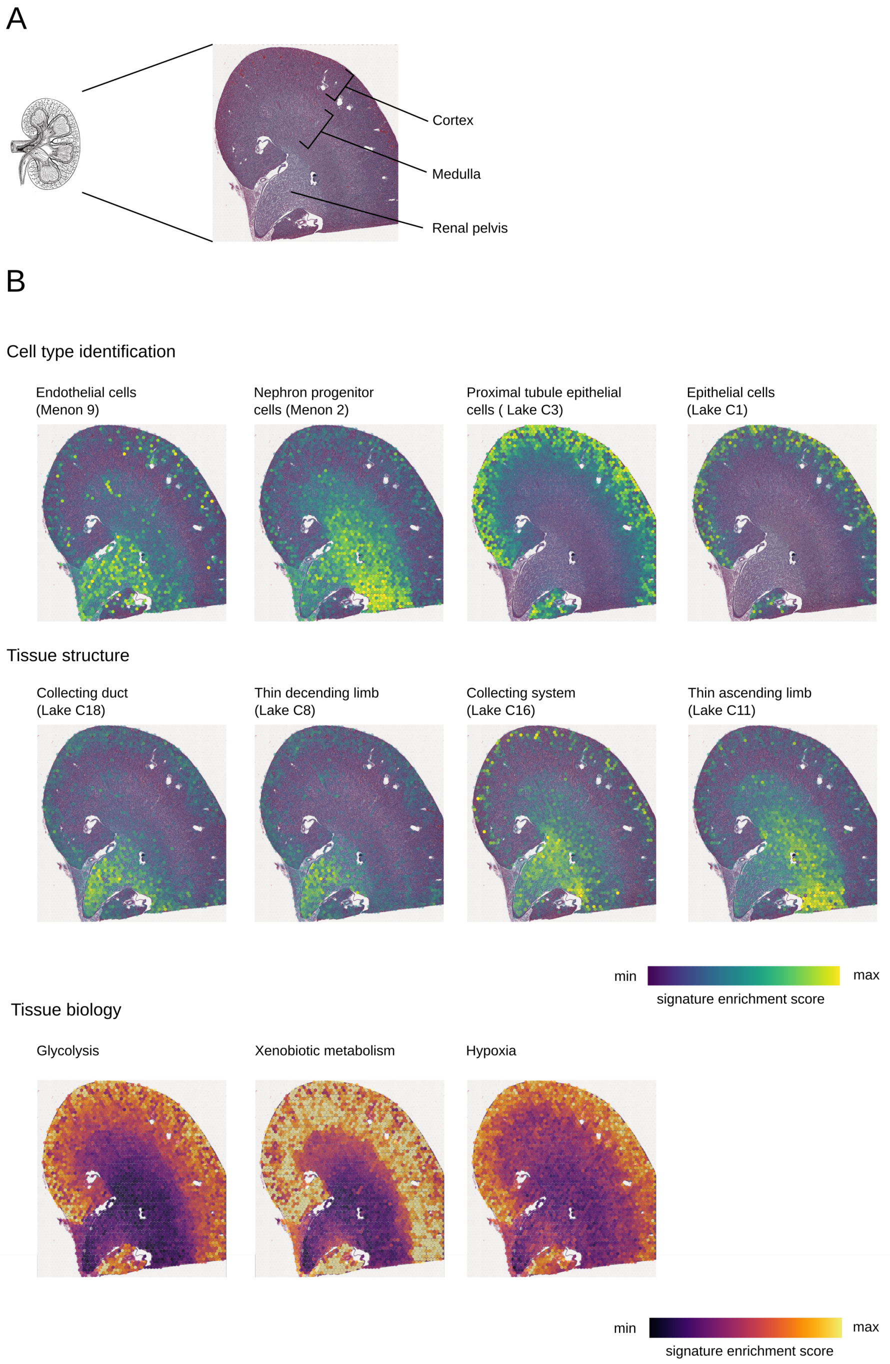

Supplementary Figure 5. The mouse kidney dataset was downloaded from 10x Genomics website. The tissue was stained with hematoxylin and aqueous eosin (HE) (A). Data were normalized using Seurat[3], and pathways from MSIgDB[2] were scored using Single-cell Explorer Scorer[1]. Using Single-Cell Spatial Explorer, signatures of cell types, tissue structure (Viridis gradient heatmap) and tissue biology (Inferno gradient heatmap) are visualized through a min-max scale and opacity threshold (B).

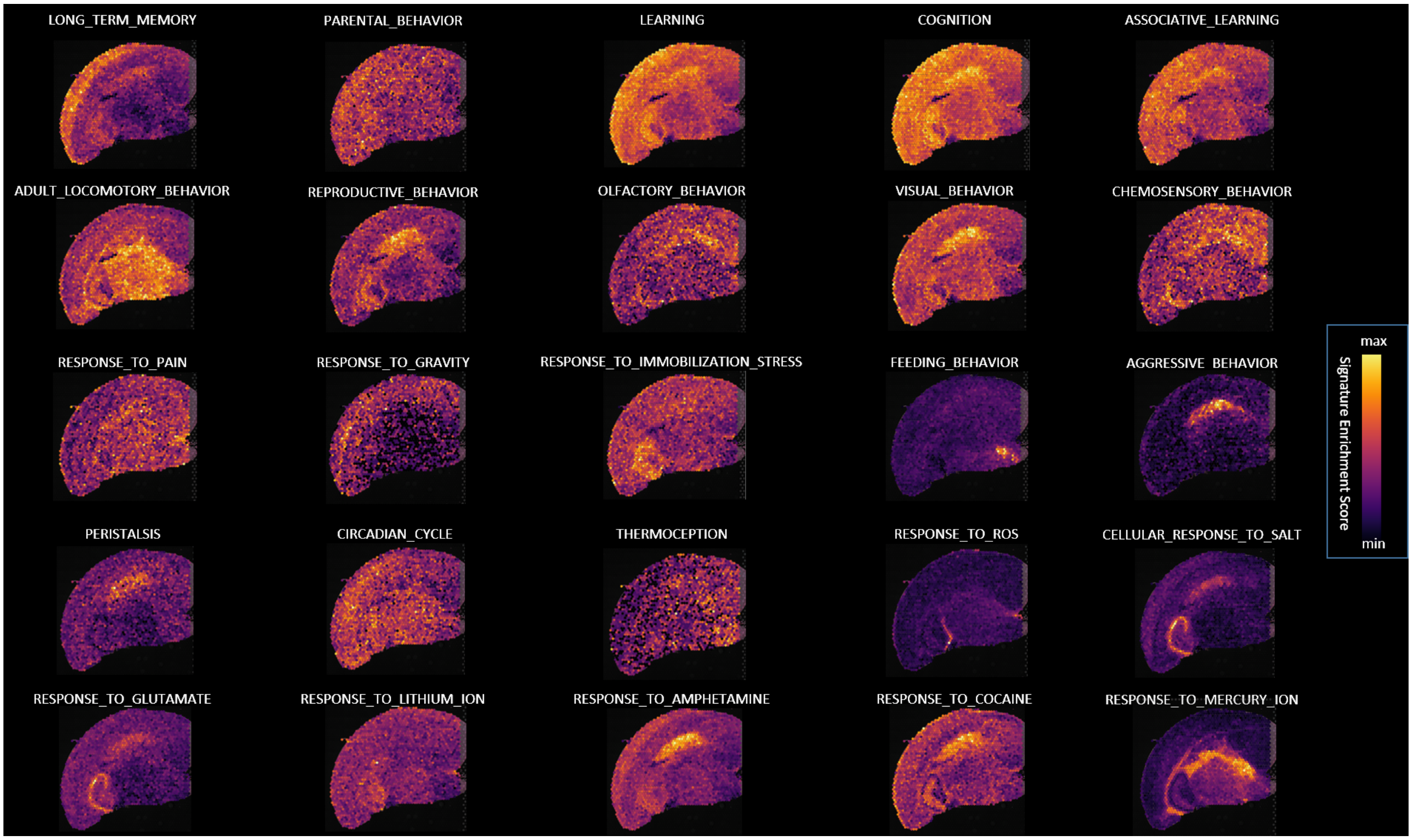

Supplementary Figure 6. The mouse brain dataset was downloaded from 10x Genomics website. Data were normalized using Seurat[3], and signatures from MSIgDB[2] were scored using Single-cell Explorer Scorer[1]. Using Single-Cell Spatial Explorer, the specified behavioral signatures from GO-BP are visualized (Inferno gradient heatmap) through a min-max scale without opacity threshold .

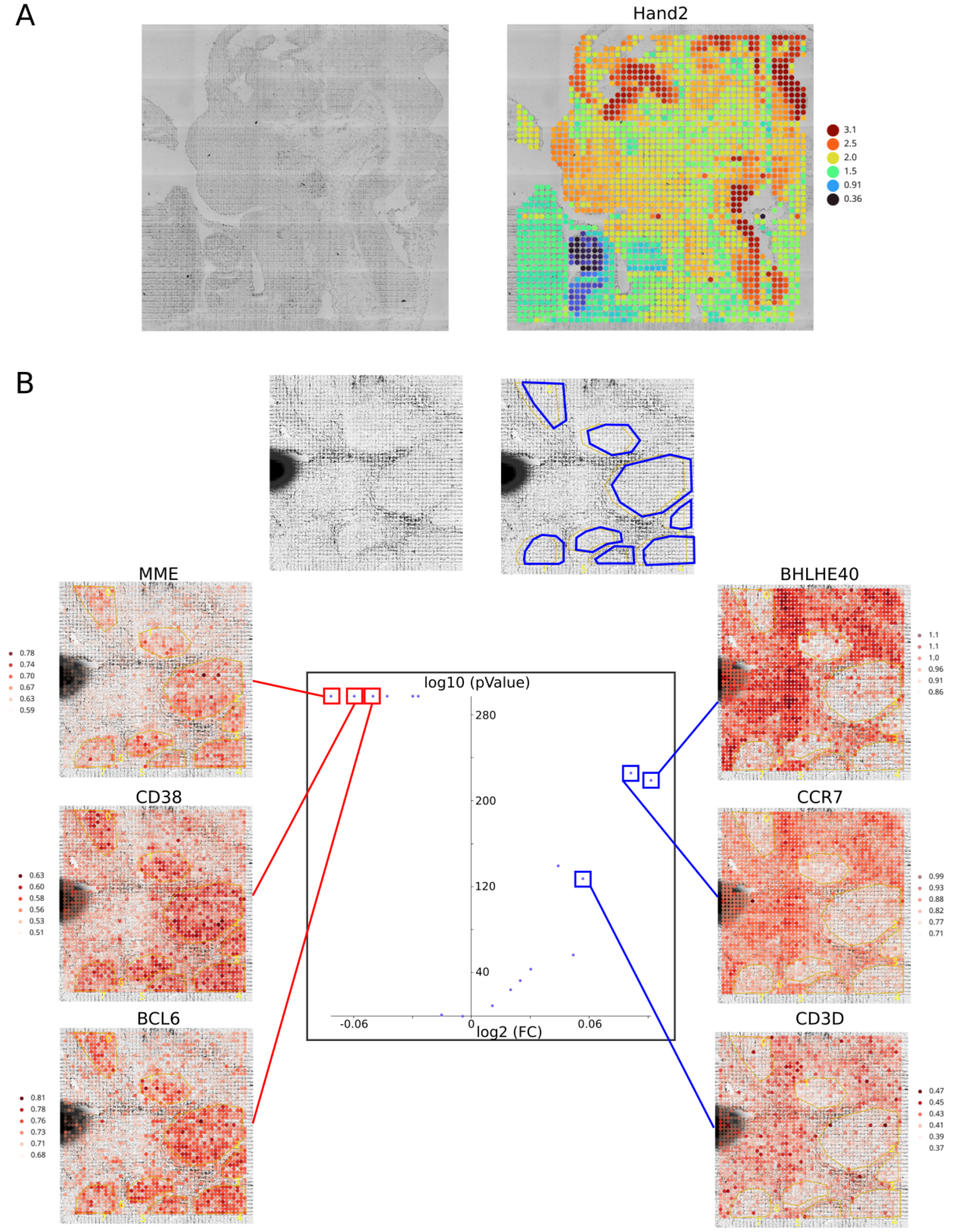

Supplementary Figure 7. Analysis of spatial ATAC-seq experiment. A: A mice sample named "ME11 H3K27me3 50um" (GSM5028434) was downloaded and data were processed as described in[4] to obtain a Seurat object. Then, genes scores were exported as a matrix, merged with spatial coordinate using the scExplorer Merger (supplementary Fig. 2) and visualized in scSpatial Explorer. B: Human tonsil sample (GSM528388) was downloaded and data were processed as described in[5], to obtain a Seurat object. Then, genes scores were exported as a matrix, merged with spatial coordinate using the scExplorer Merger (supplementary Fig. 2) and analysed in Single-Cell Spatial Explorer. Gates drawn in[5] can be easily reproduced (up right) with Single-Cell Spatial Explorer lasso and polygon drawing tools, and differential analysis of gates *versus* other areas was performed (center). The spatial visualisation of 3 up-regulated genes and 3 down-regulated genes are shown.

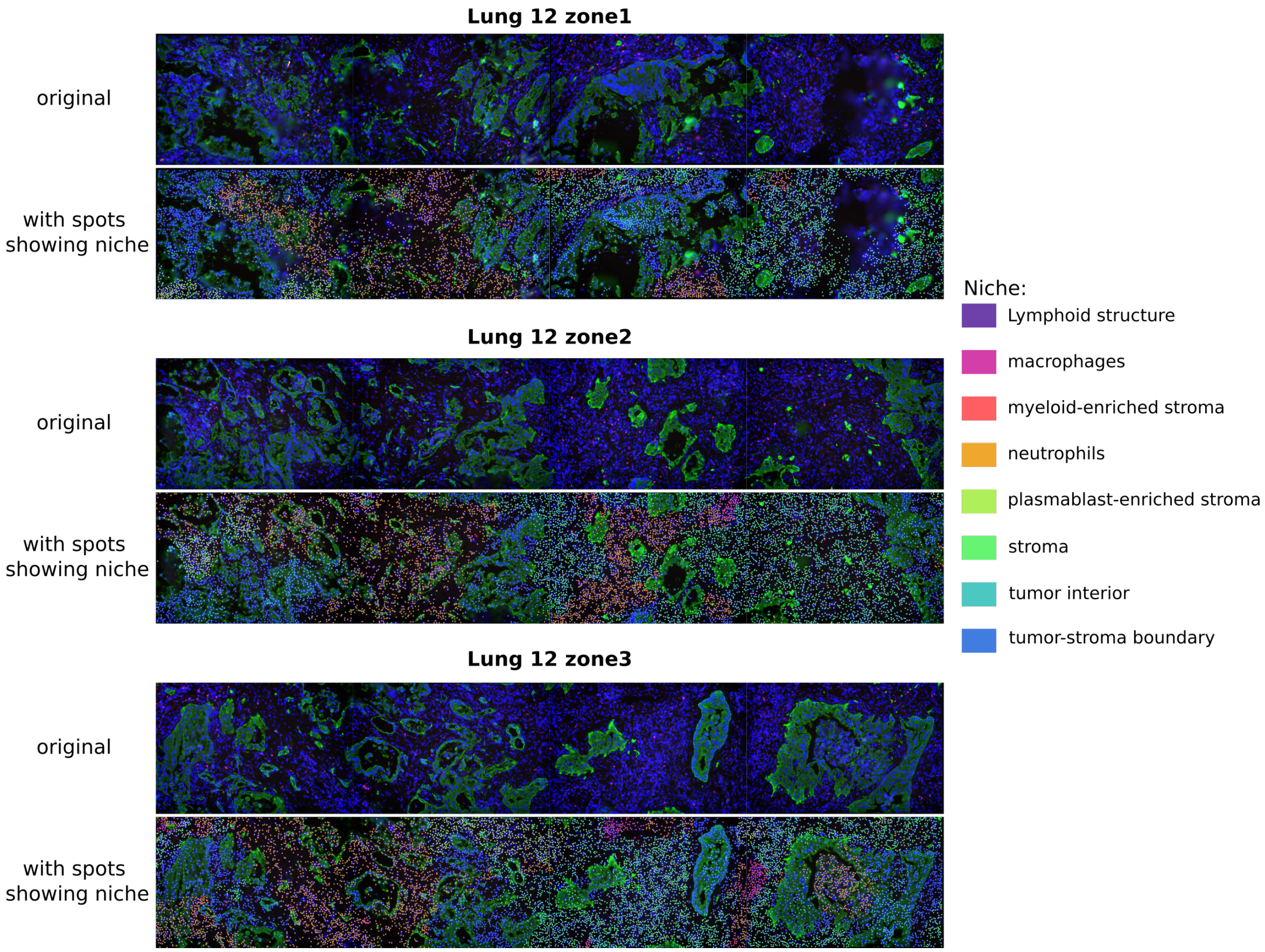

Supplementary Figure 8. Spatial analysis of lung tissue by CoxMx technology displayed in Single-Cell Spatial Explorer. The Giotto object provided by Nanostring was downloaded from the Nanostring website and metadata were exported. Three different zones are shown with the original image (up) and spots superimposed to the original image (down). The colors of the spots showing the type of niche are similar to the original analysis.

Software/Website	Single-Cell Spatial Explorer	Giotto Viewer	ST viewer
Open source	yes	yes	yes
cost	free	free	free
Installation	compiled binary (Linux, Mac, Windows)	R and Python modules	compiled binary (Mac, Windows) (C++, R, Qt) linux source
Cross platform	Linux, Mac, Windows	Linux, Mac, Windows	Linux, Mac, Windows
data format	TSV	Giotto Analyzer	TSV
Gates	unlimited	1	unlimited
Data table filtering by gates	yes – unlimited number of tables	?	no
Clusters	Image	Image	scatter plot and image
Expression	Image	Image	scatter plot and image
Expression layers	1	?	1 or more
Gates content 2D plot	yes	no	yes
Gates comparison	yes (volcano)	no	yes (volcano, correlation, PCA)
Import/export gates	yes	no	no
Filter tables	yes	no	no
variable cell opacity	yes	?	yes
cell opacity gradient	yes	no	no
Import/export cells	both	export	no
Expression slide show	yes	no	no
Test files	yes	no for the viewer	yes
Documentation	PDF manual	functions reference pages	Readme page on github. PDF in Bioinformatics website
Video	yes	yes (5)	no

Appendix 1 Table 1: continue on the next page

Appendix 1 Table1: continued from previous page

Software/Website	Vitessce	Seurat	Loupe browser	Squidpy
Open source	yes	yes	no	yes
cost	free	free	free: 10xGenomics customers	free
Installation	R, Python, JavaScript	R package	compiled binary	python
Cross platform	Linux, Mac, Windows	Linux, Mac, Windows	Windows, Mac	Linux, Mac, Windows
data format	json, Zarr, h5ad, loom, Seurat...	tsv, H5ad, 10x folder	.cloupe (proprietary)	AnnData, 10x folder
Gates	unlimited	No graphical tool	1 gate	unlimited, annotation only
Data table filter by gates	no	Filtering table by numeric criteria	no	through python only
Clusters	Image and scatter plot	Image and scatter plot	Image and scatter plot	Image and scatter plot
Expression	Image and scatter plot	Image and scatter plot	Image	Image and scatter plot
Expression layers	1 or more	1	1	1
Gates content 2D plot	yes	No graphical tool but R tools available	no	no
Gates comparison	no	No graphical tool, R tools available	yes (heatmap, violin)	yes (heatmap)
Import/export gates	no	no	no	no
Filter tables	no	No graphical tool, R tools available	no	no
variable cell opacity	yes	yes	yes	yes
cell opacity gradient	no	yes	no	no
Import/export cells	import cell sets	yes (no graphical tool)	export	export
Expression slide show	no	yes	no	no
Test files	yes	yes	yes	yes
Documentation	on line tutorial	on line tutorial	Online tutorials	Online tutorials
Video	one line demos with data sets	yes	promotional videos	Yes (6)

Appendix 1 Table1: spatial transcriptomics softwares comparison

Supplementary discussion

1 Memory footprint

Memory usage was a priority in the development of Single-Cell Spatial Explorer. Indeed, spatial transcriptomics is constantly evolving and the resolution and the number of cells increases rapidly. For most functions, Single-Cell Spatial Explorer does not load the whole dataset in RAM but loads only a needed subset. A large data set will increase hard disk usage and slow down the software; but it will still be possible to display it with an adequate amount of RAM. Single-Cell Spatial Explorer RAM footprint analysis with memusage (see supplementary fig. 1) for 2798×202 dataframe and 2798×9885 dataframe, shows a memory usage $< 400MB$. This memory footprint is very low, so Single-Cell Spatial Explorer does not require a powerful computer at least for the current resolution of spatial transcriptomics.

2 Compatibility with ImageJ/Fiji

This macro was used to export the regions of interest : Macro ImageJ/Fiji to export Region Of Interest (ROI) coordinate from ROI manager:

```
for (i=0 ; i<roiManager("count"); i++) {
    roiManager("select", i);
    roiManager("Set Color", "yellow");
roiManager("Set Line Width", 0);
    saveAs("Results", "MyFolder/XY_
    Coordinates_"+i+".csv");
}
```

3 Comparison with existing softwares

Seurat [3] is a R package designed for single-cell RNA-seq data exploration. It is a R package able to do pre-processing tasks (such as quality controls, data normalisation, samples aggregation and batch effect correction), data analysis (reduce the dimensionality with PCA/t-SNE/UMAP, cluster analysis, differential gene expression, ...) and data visualisation. Seurat is a very powerful tool targeting users with programming knowledge such as bio-informaticians with a solid R expertise.

The Giotto[6] package consists of two modules, Giotto Analyzer and Viewer, which provide tools to process, analyze and visualize single-cell spatial expression data. It is compatible with 9 different state-of-the-art spatial technologies, including in situ hybridization (seqFISH+, merFISH, osmFISH), sequencing (Slide-seq, Visium, STARmap) and imaging-based multiplexing/proteomics (CyCIF, MIBI, CODEX). Data analysis is performed first in Giotto Analyzer using command lines and exported then to Giotto viewer. A solid experience in R is needed to install Giotto since many R and Python packages are needed and some dependencies are not listed in the installation note [1].
In the Giotto Analyzer, the image alignment should be done by trial and error: the place and the zoom of the image are controlled by numerical parameters. Giotto Analyzer can filtrate genes ans cells for quality controls. At the time of the manuscript, the link to the Giotto viewer was broken and the documentation "How to switch between Giotto Analyzer and Viewer?" was not available with the status "work in progress".

ST viewer[7] is a compiled software to perform analysis and visualization of spatial transcriptomic datasets. The ST viewer enables users to visualize the location of one or multiple genes in real time in a stand-alone desktop application. ST viewer can filter and normalize data with DESeq2, reduce the dimensionality with t-SNE or PCA and compute clusters. A binary for linux is not available. Unfortunately we were not able to test ST viewer on Windows since it does not start. This issue might be related to the R version though compatible versions are not documented and remain unanswered in the dedicated forum. We opened an issue during manuscript preparation. The Mac version need QT recompilation which is not accessible to the average user.

Vitessce[8] (Visual integration tool for exploration of spatial single cell experiments) is an open-source interactive visualization framework for exploration of multimodal and spatially resolved single-cell data; It presents a modular architecture compatible with transcriptomic, proteomic, genome-mapped, and imaging data types. In contrast to scSpatial Explorer, Vitessce can be used as a standalone web application or in Python or R environments. This software has nice online demos with 8 data sets.

Loupe Cell browser is a dedicated visualization and analysis tool for scRNAseq developed for analysing scRNAseq datasets produced by 10xGenomic platforms. It allows importing datasets and visualizing custom projections of either gene expression

[1] such as openssl, libcurl4-openssl-dev, libxml2-dev, libssl-dev, libgmp3-dev, libgsl-dev, libmagick++-dev

or antibody-only datasets, across t-SNE or UMAP computed by the Cell Ranger pipeline. Loupe Cell browser also provides Moran's I for pattern analysis of single gene expression level views across the image. Despite its ease of use however, this tool does not provide signature visualizations, nor the corresponding image pattern analyses.

SquidPy[9] is a Python library dedicated to the analysis and visualisation of single-cell spatial transcriptomics datasets. The installation as only been tested on Linux, and requires some basic Python knowledge. This library is used after Scanpy QC & normalisation of the spatial dataset. This powerful tool gives a lot of analytic possibilities, such as cluster annotation, cluster features computation, neighborhood enrichment or even ligand-receptor interaction analysis. This tool also offers the possibility to integrate other libraries to perform complementary analysis. SquidPy has an easy-to-use graphical interface, but its main analytic features are only available through command line, eventually preventing some users to explore their data to the fullest.

Commercial softwares, such as BBrowser or Partek Flow are powerful analysis and visualisation softwares for single cell transcriptome analysis and spatial transcriptomics. However, since they are proprietary, not open source, and their cost is not specified in their website, they could not be tested here.

So currently, quite a few existing open source tools allow to visualize single cell spatial transcriptomics data (see software comparison in supplementary Table 1). We found two softwares, Loupe and STviewer, which are free of charge[2] and provide a ready-to-use binary executable like Single-Cell Spatial Explorer. Unfortunately we had issues with the compiled binaries of STviewer and Loupe interoperability is very far from what is possible with Single-Cell Spatial Explorer. By being mainly dedicated to data visualization, Single-Cell Spatial Explorer is mostly recommended for biologists, pathologists and biomedical users that are not familiar with R or command line tools. In conclusion, Single-Cell Spatial Explorer is currently the best alternative for users who want a software which works out of the box, without tedious installation.

[2] Loupe is free of charge for 10x Genomics customers.

References

1. Pont, F., Tosolini, M. & Fournié, J.-J. Single-Cell Signature Explorer for comprehensive visualization of single cell signatures across scRNA-seq datasets. *Nucleic acids research* **47**, e133–e133, DOI: 10.1093/nar/gkz601 (2019).
2. Liberzon, A. *et al.* The molecular signatures database (msigdb) hallmark gene set collection. *Cell systems* **1**, 417–425, DOI: 10.1016/j.cels.2015.12.004 (2015).
3. Butler, A., Hoffman, P., Smibert, P., Papalexi, E. & Satija, R. Integrating single-cell transcriptomic data across different conditions, technologies, and species. *Nat. biotechnology* **36**, 411–420, DOI: 10.1038/nbt.4096 (2018).
4. Deng, Y. *et al.* Spatial-cut&tag: spatially resolved chromatin modification profiling at the cellular level. *Science* **375**, 681–686 (2022).
5. Deng, Y. *et al.* Spatial profiling of chromatin accessibility in mouse and human tissues. *Nature* **609**, 375–383 (2022).
6. Dries, R. *et al.* Giotto, a toolbox for integrative analysis and visualization of spatial expression data. *bioRxiv* DOI: 10.1101/701680 (2020).
7. Fernández Navarro, J., Lundeberg, J. & Ståhl, P. L. ST viewer: a tool for analysis and visualization of spatial transcriptomics datasets. *Bioinformatics* **35**, 1058–1060, DOI: 10.1093/bioinformatics/bty714 (2018).
8. Keller, M. S. *et al.* Vitessce: a framework for integrative visualization of multi-modal and spatially-resolved single-cell data. *OSF Prepr.* DOI: 10.31219/osf.io/y8thv (2021).
9. Palla, G. *et al.* Squidpy: a scalable framework for spatial omics analysis. *Nat. Methods* **19**, 171–178, DOI: 10.1038/s41592-021-01358-2 (2022).
